# Grass Cell Walls: A Story of Cross-Linking

**DOI:** 10.3389/fpls.2016.02056

**Published:** 2017-01-18

**Authors:** Ronald D. Hatfield, David M. Rancour, Jane M. Marita

**Affiliations:** U.S. Dairy Forage Research Center, USDA-Agricultural Research ServiceMadison, WI, USA

**Keywords:** grasses, cell walls, ferulates, *p*-coumarates, lignin, cross-linking, glucuronoarabinoxylans

## Abstract

Cell wall matrices are complex composites mainly of polysaccharides, phenolics (monomers and polymers), and protein. We are beginning to understand the synthesis of these major wall components individually, but still have a poor understanding of how cell walls are assembled into complex matrices. Valuable insight has been gained by examining intact components to understand the individual elements that make up plant cell walls. Grasses are a prominent group within the plant kingdom, not only for their important roles in global agriculture, but also for the complexity of their cell walls. Ferulate incorporation into grass cell wall matrices (C3 and C4 types) leads to a cross-linked matrix that plays a prominent role in the structure and utilization of grass biomass compared to dicot species. Incorporation of *p*-coumarates as part of the lignin structure also adds to the complexity of grass cell walls. Feruoylation results in a wall with individual hemicellulosic polysaccharides (arabinoxylans) covalently linked to each other and to lignin. Evidence strongly suggests that ferulates not only cross-link arabinoxylans, but may be important factors in lignification of the cell wall. Therefore, the distribution of ferulates on arabinoxylans could provide a means of structuring regions of the matrix with the incorporation of lignin and have a significant impact upon localized cell wall organization. The role of other phenolics in cell wall formation such as *p*-coumarates (which can have concentrations higher than ferulates) remains unknown. It is possible that *p*-coumarates assist in the formation of lignin, especially syringyl rich lignin. The uniqueness of the grass cell wall compared to dicot sepcies may not be so much in the gross composition of the wall, but how the distinctive individual components are organized into a functional wall matrix. These features are discussed and working models are provided to illustrate how changing the organization of feruoylation and *p*-coumaroylation could lead to differing cell wall properties.

## Introduction

Annual and perennial grasses play a vital role in agriculture by providing feedstuffs for animals in the forms of fresh forage (grazing) and preserved forage (silage and hay). The grains harvested from grasses comprise an important food source for both animals and humans. In addition, perennial grasses play a pivotal role in stabilizing soils and minimizing soil erosion especially in areas that are considered marginal lands. Understanding the functional roles of cell walls as it relates to plant growth, development, and responses to the environment would be useful and important for agronomic productivity and utilization. Increased knowledge of cell walls would result in greater and more efficient utilization as feedstuff for ruminants as well as improved sources of biomass for bioenergy.

### Structural polysaccharides (xylans)

In general, plant cell walls have similar main structural features. They all contain a cellulosic backbone imbedded in a variable matrix made up of structural polysaccharides (hemicellulosic polysaccharides and pectins), lignin, and proteins (both structural and metabolic). The proportions and specific types of polysaccharides within the major groups can vary among species of plants providing a general fingerprint for a given species. This is complicated by the variable amounts of different components depending upon the stage of development and the organ- and cell-type source of the cell wall.

**Figure 1:**
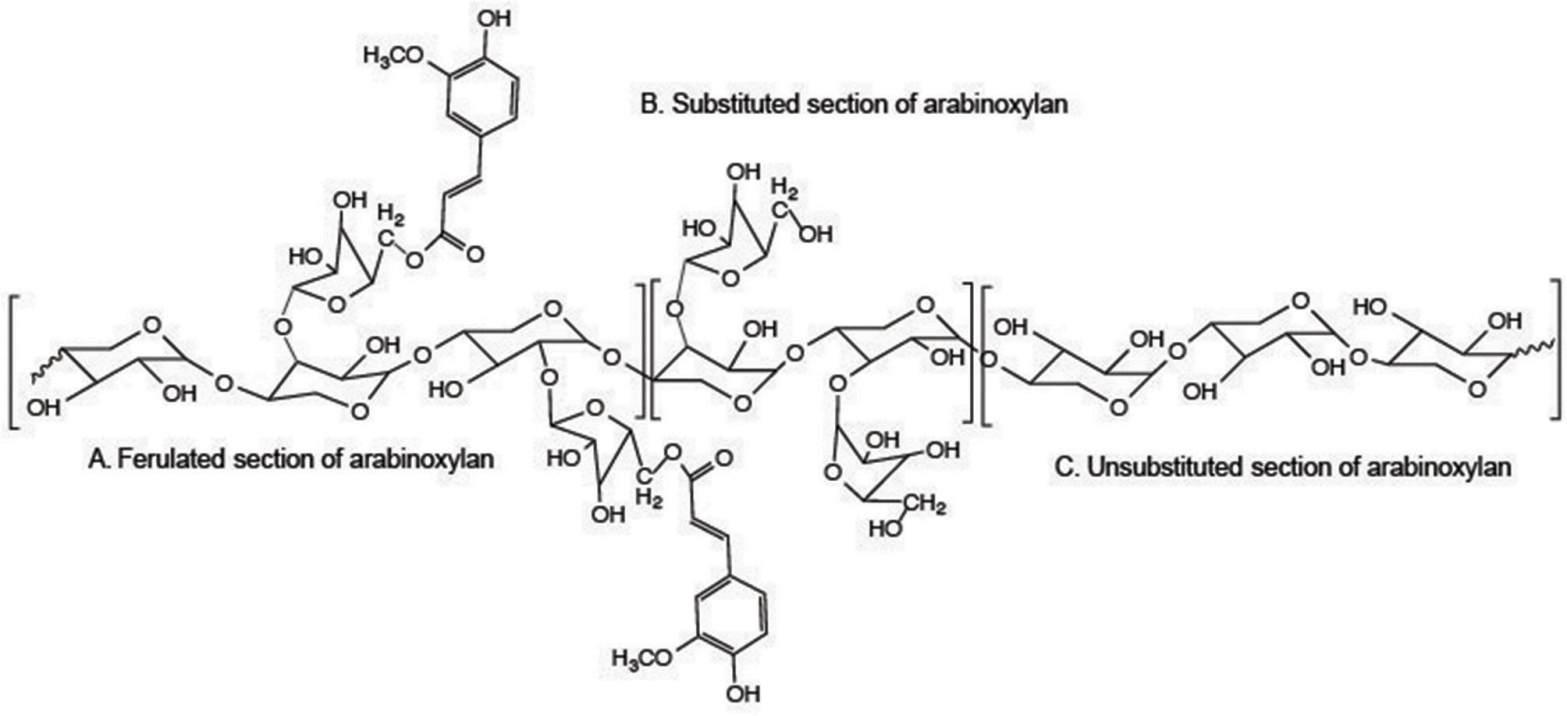
**Chemical molecular model of grass xylan**. Molecular models of grass xylan domains including **(A)** regions of arabinoxylan substituted with ferulic acid ester-linked to the 5-OH of arabinofuranose, **(B)** β-1,4-xylose substituted with α–1,2 and/or α–1,3-arabinofuranose for arabinoxylan, and **(C)** unsubstituted ß-1,4-xylose.

In grass cell walls the major hemicellulosic polysaccharide is the xylan. Typically grass xylans make up between 20 and 30% of the total cell wall. The non-xylan, non-cellulosic polysaccharides comprise between 10 and 15% of the cell wall. Grass xylan composition differs from dicot xylan in that it is substituted with arabinofuranose (Ara*f*) and ester-liked hydroxycinnamates (Figure 1; Carpita, 1996; Pauly et al., 2013; Rennie and Scheller, 2014). Ara*f* substitution of the β-(1,4)-xylose backbone may be α-(1,2) or α-(1,3)-linked. Additionally, the β-(1,4)-xylose backbone can be substituted with α-(1,2)-glucuronic acid to form glucuronoarabinoxylans (GAX) containing both arabinosyl and glucuronosyl substitutions on the β–1,4 xylan backbone. Arabinosyl substitution of grass xylan can vary from molar ratios of 1:2 Ara:Xyl to levels of 1:20 or 1:30 depending upon the maturity of the specific grass and tissue being evaluated. Substitution patterns on the xylan backbone dictate how strongly they can hydrogen bond to other wall polysaccharides, mainly cellulose and to other xylans, influencing structural properties of the wall (Ebringerova and Heinze, 2000).

Characterization of the xylan reducing end structure in monocots lags dicot systems. In dicots and gymnosperms, a specific reducing end sequence (4-β-D-Xyl*p*-(1,4)- β-D-Xyl*p*-(1,3)- α-L-Rha*p*-(1,2)- α-D-Gal*p*A-(1,4)-D-Xyl*p*) has been found (Pena et al., 2007). Recently, targeted identification of the reducing end structure from wheat endosperm xylans indicate a significantly different sequence comprising of linear 1,4-β-D-Xyl*p* mono-substituted with an 1,3-α-L-Ara*f* at the terminal or penultimate Xyl*p* and/or an 1,2-α-D-GlcA on the terminal Xyl*p* residue (Ratnayake et al., 2014).

**Figure 2:**
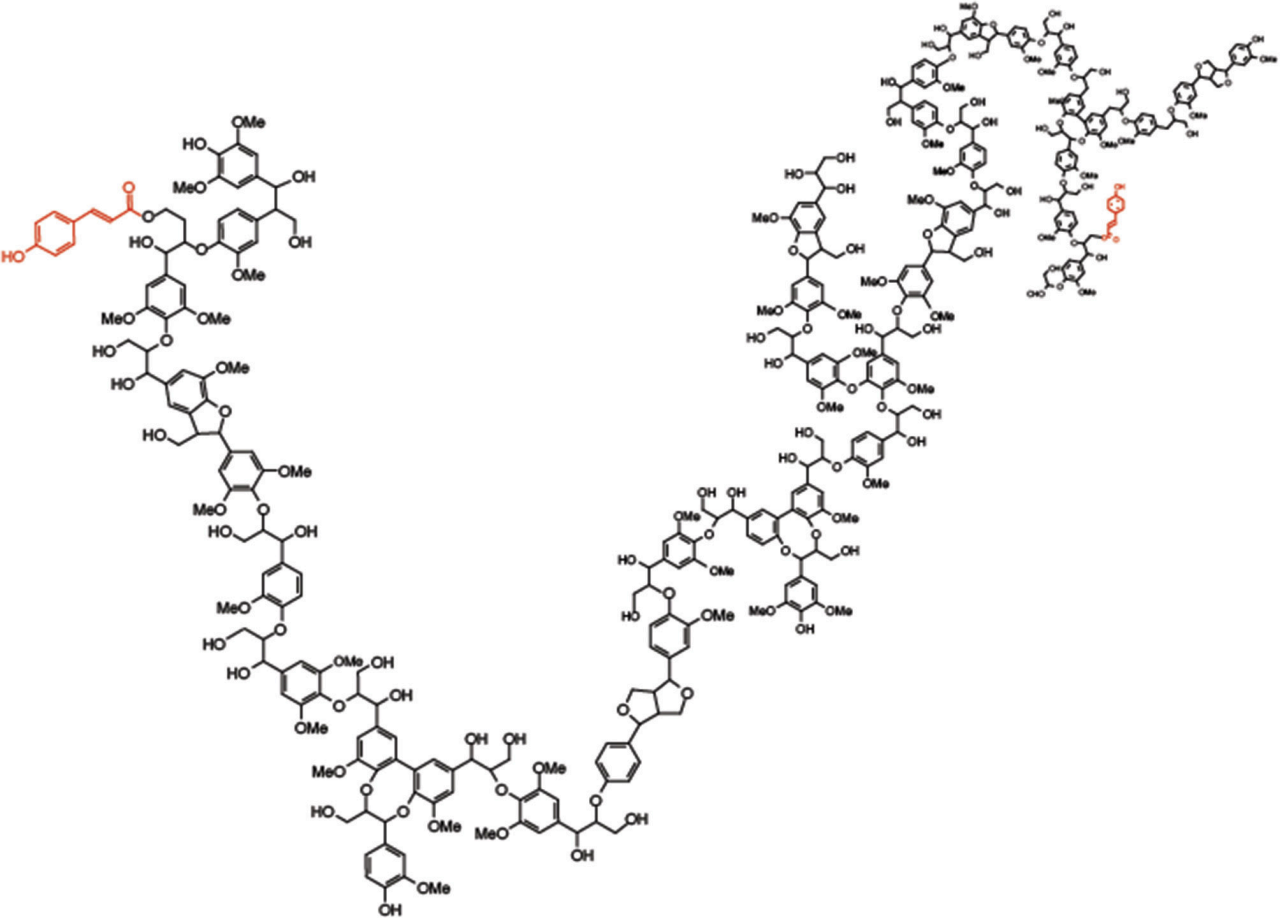
**Molecular model of grass lignin**. Molecular model of grass lignin highlighting the diverse linkages contained within the polymer including *p*-coumarate ester-linked (red) to sinapyl subunits.

Xylans in grasses have another unique structural feature when compared to dicot xylans: the addition of ferulic acid (FA) and, to a lesser extent, *p*-coumaric acid (*p*CA) to the α-(1,3)-Ara*f* residues (Ishii, 1997). Ferulic acid and *p*CA are attached by ester linkages to the C-5 carbon of arabinofuranosyl (Ara*f*) residues (Figure 1). Not every arabinosyl residue contains a ferulate and the exact placement along the xylan backbone remains unknown at this time. There is continued debate as to the placement of Ara*f* residues to form uniform repeating structures (Faik, 2010). Detailed structural studies using purified endoxylanases followed by structural characterization of the purified released oligosaccharides indicated uniformity of these carbohydrate fractions (Zeng et al., 2008). Carpita (1984b) identified a highly substituted GAX in maize that was structurally related to other GAX polysaccharides from maize with a much lower level of substitution on the xylan backbone. It was speculated that GAX biosynthesis may initially occur in the highly substituted form and enzymatically modified once secreted into the cell wall. Such a mechanism would help with manipulation of the xylan into a particular location followed by removal of the arabinosyl substitutions to promote hydrogen bonding to other xylans and/or cellulose (Figure 2). Arabinofuranosidase activity needed to remove the side chain substitutions may be limited by the presence of ferulates attached to some of the Ara*f* residues. This action could result in what appears to be a relatively uniform structure but is controlled post synthesis as opposed to controlling the appropriate glycosyltransferases.

The presence of ferulates on Ara*f* residues varies from grass species to grass species and also within different tissues. In mature maize the ratio of FA:Ara*f* can be nearly 1:1 for pith tissues and 1:2 for the rind portion of the stem (Hatfield and Chaptman, 2009). In *Brachypodium* (Rancour et al., 2012) the FA:Ara*f* ratio varied depending upon the stage of development and plant tissue. In mature stems FA:Ara*f* was 1:2.4 while expanding stems were 1:3.1, mature sheath material was 1:3.9 and expanding sheath was 1:10.7 and leaf material remained relatively constant at 1:4.7 FA:Ara*f*. There is other data in the literature but it is difficult to draw conclusions concerning the FA:Ara*f* as it is not always clear if the FA analyses accounted for both ester linked and ether linked or whether FA dimers were included in the analyses. However, these data do not address if the observed variation in the FA:Ara*f* ratio is controlled at the time of FA-AX synthesis or is the result of post-synthesis modification by appropriate hydrolytic enzymes, such as arabinofurnanosideases.

### Ferulates and *p*-coumarates

The majority of the Poaceae family (true grasses) incorporates ferulic acid (FA) and *p*-coumaric acid (*p*CA) into their cell walls. Harris and Hartley (1980), Harris et al. (1980) identified the acylation of plant cell walls with both *p*-hydroxycinnamates. It is widely accepted that grass cell walls are uniquely cross-linked by ferulates to form diferulates and to some extent *p*-coumarate cyclodimers (Fry, 1986; Ford and Hartley, 1990; Quideau and Ralph, 1997; Ralph et al., 1998; Hatfield et al., 1999a). This cross-linking extends to coupling carbohydrate fractions (GAX) to lignin. The degradability of grass cell walls can be affected by manipulating the degree of cross-linking and lignification (Ford and Elliott, 1987; Jung et al., 1992; Grabber et al., 1998a). This degradability is important for utilization as nutrient carbohydrate sources in livestock systems and in feedstock for bioenergy production systems.

Identification of phenolic acids in plant cell walls dates back several decades. Since their identification, much work has been devoted to defining their roles within cell wall matrices. Early on it was shown that ferulates incorporated into grass cell walls could form cross-links thus coupling together carbohydrate polymers, principally GAX (Ishii and Hiroi, 1990; Ishii, 1997). For a long time it was believed that only 5-5-diferulate formed cross-links within cell wall matrices (Markwalder and Neukom, 1976; Neukom and Markwalder, 1978). Subsequent work clearly demonstrated that several types of ferulate dimers could be formed from free radical mediated coupling reactions (Ralph et al., 1994; Grabber et al., 2000) as well as trimers and tetramers (Bunzel et al., 2005). Ferulates can become linked to lignin through the same type of free radical meditated cross-linking to form a carbohydrate and lignin complex covalently linked together in grass cell walls. It is clear the role of ferulates is to cross-link not only the carbohydrate fraction (mainly GAX) of the cell wall matrices but to also form covalent linkages to growing lignin polymers during the lignification process (Quideau and Ralph, 1997; Bunzel et al., 2005). Structural evidence suggests that ferulates can act as nucleation sites for lignin formation (Ralph et al., 1993, 1995; Grabber et al., 1998b). One can envision lignification occurring and spreading out from nucleation sites of feruloylated cell walls. Initially, lignification from these numerous sites could form small separate pools of lignin polymers within grass walls. This may explain why grass lignin especially in immature plants is reasonably alkaline soluble compared to many dicots. The advantage to the grass is in maintaining a more flexible stem particularly at early stages of development.

The significant amounts of *p*CA on lignin in these same grass cell walls and the functional role *p*-coumaroylation plays within the cell wall remains unclear. There are reports of *p*CA being ester linked to GAX just like FA, yet this level is much lower (1:15) compared to FA (Mueller-Harvey et al., 1986). Other studies examining corn failed to identify detectable levels of *p*CA attached to arabinose in the stem tissues using methods based on mild acid hydrolysis to break the C1 arabinosyl linkage to xylans releasing *p*CA-arabinose and FA-arabinose conjugates (Myton and Fry, 1994; Marita et al., 2003; Hatfield et al., 2008b). In corn stems as well as other grasses, *p*CA incorporation into cell walls as part of the lignin fraction seems to be a preferred. Recent reports have indicated substantial increases of ester-linked *p*CA incorporation in place of FA on arabinoxylan can be achieved in rice through the over-expression of OsAt10, a BAHD acyl-CoA transferase (Bartley et al., 2013). This replacement of FA with *p*CA resulted in saccharification improvements of 20–40% without affecting plant development nor lignin content or composition.

Corn (*Zea mays*) has some of the highest esterified-*p*CA levels along with grain sorghum (*Sorghum bicolor*) (30–38 g kg^−1^ cell wall). Other C4 grasses like switchgrass, big bluestem, and little bluestem have significant levels (12–15 g kg^−1^ CW), but are usually less than half the levels found in grain sorghum or corn and are similar to the levels seen in C3 type grasses (Hatfield et al., 2009). One explanation for greater quantities in corn and grain sorghum over other grass species is independent of the unique factors classifying them as C4 or C3 type grass. Such differences could simply be related to the overall size of the plants. Lignin isolated from taller grasses with larger diameter stems results in a greater degree of *p*-coumaroylation. To clearly determine the validity of this a more diverse cross section of grasses needs to be examined across multiple locations and environments.

One proposed role of *p*CA in grass cell walls is as a radical transfer mechanism to help in the formation of lignin monomer radicals especially sinapyl alcohol (SA) (Takahama and Oniki, 1996, 1997; Hatfield et al., 2008a). Incorporation of *p*CA into the wall matrix occurs through the intracellular attachment of *p*CA to SA or coniferyl alcohol (CA) residues via esterification. In grasses such as corn, the cell wall peroxidases do not oxidize SA as rapidly as CA, FA, or *p*CA. However, in its oxidized state, *p*CA can rapidly transfer a free radical to SA resulting in rapid radical mediated cross-coupling reactions between SA residues or SA and CA (Ralph et al., 2004a). Attaching *p*CA to monolignols especially SA residues insures that *p*CA is in the same location within the wall matrix as the polymerizing monolignols. As such, one might expect in grasses or tissues with higher levels of *p*CA, there would be higher overall lignin content within the cell wall matrix. A comparison of C3 and C4 grasses indicates on a cell wall basis lignin levels were nearly the same across all grasses (155–225 g kg^−1^ CW) while *p*CA ranged from (5–37 g kg^−1^ CW). There was a 6-fold difference in *p*CA levels but only a 31–45% difference between the highest and lowest lignin content. If the role of *p*CA is to aid in the formation of lignin especially syringyl rich lignin one might expect a strong correlation between *p*CA content and total lignin. This does not appear to be the case when comparing levels across a range of different grasses (Hatfield et al., 2009), but does hold true looking within nodes/internodes of a developing corn plant, directional trends of *p*CA levels in nodes and internodes along a corn plant do correlate with corresponding lignin levels (Jung et al., 1998; Hatfield et al., 2008b). Therefore, in the current lignification model where peroxidases and/or laccases provide the oxidative capacity to plant cell walls (Vanholme et al., 2008), and the presence of phenolic compounds such as *p*CA have the potential to radicalize and be incorporated into the lignin polymer, this does not occur. Instead *p*CA acts as a radical transfer system but it does not become part of the radical mediated cross-coupling reactions that form the growing lignin polymer (Hatfield et al., 2008a). In essence, *p*CA can be recycled in this radical transfer system alternating between its oxidized and ground states during the formation of SA radicals. The *p*CA attachment to lignin is only through its ester linkage to monolignols, primarily sinapyl alcohol. Unlike ferulates that do become readily cross-linked into the growing lignin polymer, *p*CA remains bound within the cell wall matrix by this single covalent linkage, i.e., esterified to SA residues. This property of *p*CA raises two possible scenarios for its role in grass lignin (1) to act as a termination molecule for a developing lignin polymer and (2) to contribute to enhancing the linear or less reticulated nature of syringyl type lignin found in grasses.

### Lignin

**Figure 3:**
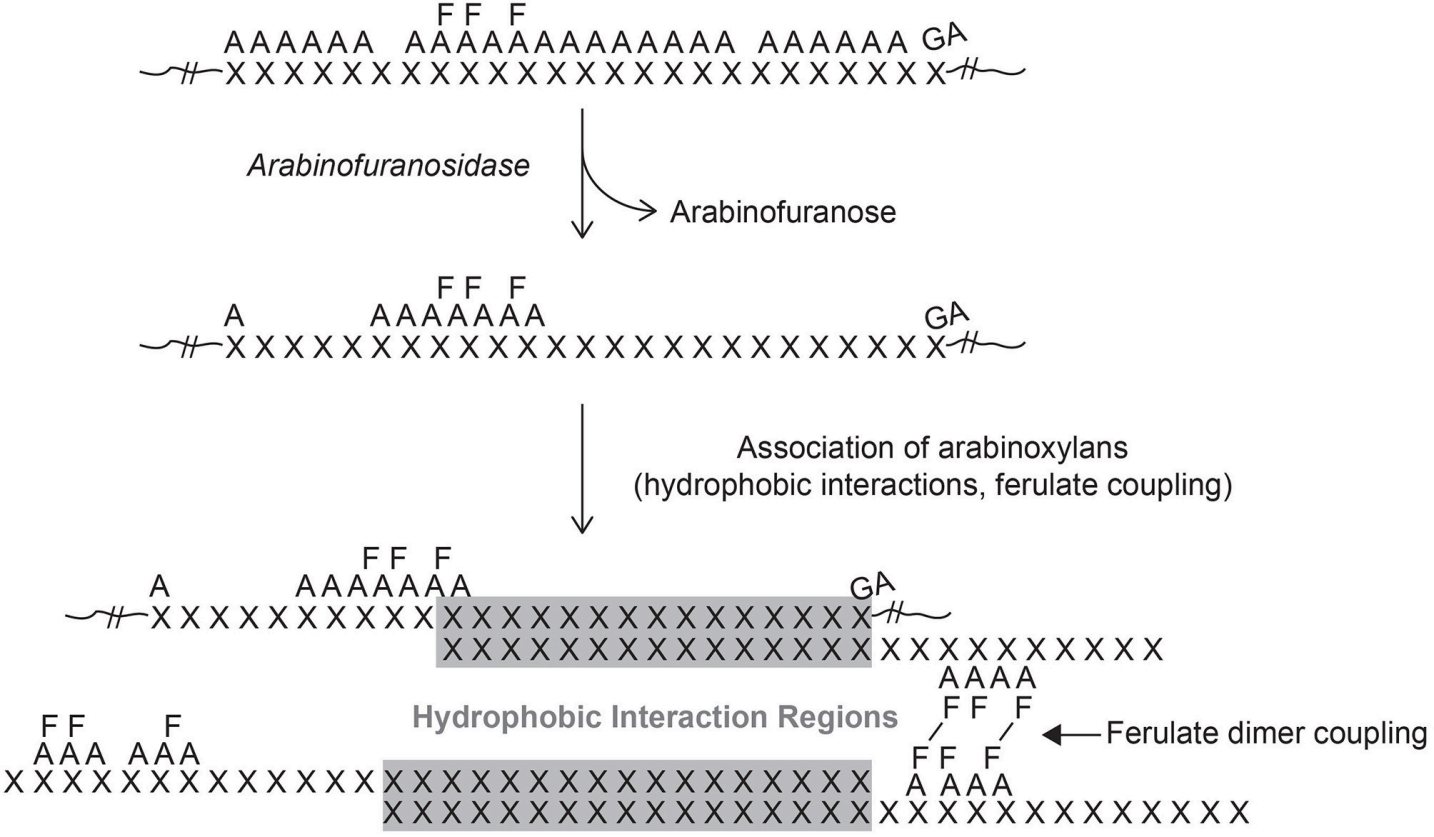
**Model of molecular interactions for grass xylans**. The presented model depicts the proposed progression of xylan development and its interactions. Xylan (X) is synthesized with a high degree of arabinose (A) substitution with regions of higher ester-linked ferulic acid (F) and those that contain glucuronic acid (GA) substitutions. Secretion into the extracellular space would provide access of arabinosidases that could remove unsubstituted arabinose units leaving linear regions of xylan exposed. These exposed xylan regions could participate in hydrogen-bonding with other exposed xylan regions and or cellulose microfibrils to stabilize cell wall architecture (gray shaded region). In addition, ferulic acids could form crosslinks to covalently stabilize interactions between opposing FA-AX polymers.

Lignin is a polymeric phenylpropanol material formed within cell wall matrices of plants. As a hydrophobic material it forces the water out of spaces in the wall matrix as it forms decreasing the flexibility and permeability. Lignin biosynthesis is controlled by developmental and environmental signals (Sarkanen and Ludwig, 1971; Sederoff et al., 1999; Vanholme et al., 2010) with many factors regulating lignin content, composition and linkage structure (Barrière et al., 2007; Li and Chapple, 2010; Shen et al., 2013). Recent work has demonstrated lignin composition and structure can be manipulated through the introduction of unique phenolic components not typically found in lignin (Elumalai et al., 2012; Vanholme et al., 2012; Tsuji et al., 2015). Traditionally the majority of genetic, genomic, and biochemical studies of lignin have focused on perennial or annual dicots (Reddy et al., 2005; Shadle et al., 2007; Wang et al., 2013; Anderson et al., 2015). Interest in forages as bioenergy resources has refocused efforts on understanding the genetic basis of lignin formation in grasses (Barrière et al., 2007; Barriere et al., 2013; Shen et al., 2013). Grass lignin polymers can be composed of three main types of units present in grass, *p*-hydroxyphenyl, guaiacyl, and syringyl units connected by aryl ether bonds (ß-*O*-4 and α-*O*-4 linkages), biphenyl ether bonds (4-*O*-5 and 5-*O*-4), and/or resistant carbon-carbon bonds (ß-5, ß-ß and 5-5) (Ralph et al., 2004b). In addition, the *p*-hydroxycinnamates, ferulic acid (FA), and *p*-coumaric acid (*p*CA), have been shown to be ester or ether linked to lignin in grasses (Figure 3).

### Cell wall proteins

The cell walls of plants are metabolically active tissues with a wide range of proteins ranging from structural components to cell wall hydrolases. Although all of these proteins play important roles in cell wall function it is not always clear what that role may be in grasses. Two general groups that are of importance are the oxidizing enzymes (peroxidases and laccases) and carbohydrate hydrolases, the latter group being important in remodeling the cell wall for expansion as well as post expansion secondary wall formation. A primary role of the peroxidases is to initiate formation of radicals in radical mediated cross-coupling reactions. In grasses, this results in the formation of ferulate dimers cross-coupling GAX as well as coupling ferulates monomers and dimers to lignin. The cell wall hydrolytic ß-glucanases are critical for the remodeling of the primary wall removing or at least releasing portions of the extensive (1,3)-(1,4)-ß-glucan network to allow cell wall expansion (Huber and Nevins, 1979; Nevins et al., 1984; Hatfield and Nevins, 1986, 1987). The other critical hydrolase is the arabinofuranosidase responsible for shaping the GAX molecules after synthesis to fit specific functional roles within the cell wall matrix (Herve et al., 2010; Sumiyoshi et al., 2013).

Though the exact distribution of proteins within the wall matrix is not clear it is likely many may be found in the cell wall free space. While others are more likely to be synthesized throughout the development of the cell wall and become incorporated into the growing wall matrix. Of particular importance are the peroxidases or laccases that are involved in the formation of cross-linked phenolics. It is likely that as the wall matrix is being formed peroxidases and/or laccases become imbedded within the matrix in an active form to initiate oxidation of phenolic compounds (monolignols, FA, *p*CA) similar to that observed in Arabidopsis (Schuetz et al., 2014). This would ensure the formation of radicals needed to facilitate cross-coupling reactions in the areas in which they will be needed. Begovic et al. (2015)

## Synthesis of wall components involved in cell wall cross-linking

### Feruloylated arabinoxylans: genes and enzymes

Identification of candidate enzymes responsible for the biosynthesis of grass feruloylarabinoxylan (FA-AX) and the genes encoding these proteins has been of great interest but remain elusive. Identification of the genes and enzymes that control FA-AX biosynthesis could lead to their manipulation in biomass production crops to improve utilization (Pauly and Keegstra, 2008). Complimentary approaches have been used and will be discussed below.

Based on the structural complexity of grass FA/*p*CA-AXs, the minimal set of enzymes needed for direct biosynthesis would include five glycosyltransferases (GTs) and two acyltransferases including (1) ß-(1,4)-xylosyltransferase (1,4-XylT), (2) ß-(1,2)-xylosyltransferase (1,4-XylT), (3) α-(1,3)-arabinofuranosyltransferase (1,3-ArafT), (4) α-(1,2)-arabinofuranosyltransferase (1,2-ArafT), (5) α-(1,2)-glucuronosyltransferase (1,2-GlcAT), and both (6) a feruloyltransferase (FAT) and (7) a *p*-coumaryltransferase (*p*CAT) (Faik, 2010; Rennie and Scheller, 2014).

Approaches taking advantage of gene expression analysis have identified candidate genes involved in grass FA-AX biosynthesis (Mitchell et al., 2007; Cao et al., 2008; Zeng et al., 2010; Bosch et al., 2011; Pellny et al., 2012; Wilson et al., 2012; Chiniquy et al., 2013; Zhang et al., 2014). For example, Mitchell et al. (2007) made a comparative analysis of expressed sequence tag (ESTs) abundance for grasses and dicots to identify carbohydrate active gene (CASy Cantarel et al., 2009) orthologs that were preferentially expressed in rice (*Oryza sativa*), wheat (*Triticum aestivum*), and barley (*Hordeum vulgare*) compared to *Arabidopsis thaliana*, soybean (*Glycine max*), *Brassica spp*., and potato (*Solanum tuberosum*). The author's hypothesis was that genes encoding FA-AX synthetic enzymes would be expressed at higher levels in monocots compared to orthologs in dicots. The analysis implicated family members of the GT43, GT47, GT61, and PF02458 families, a class of BAHD acyl transferases, as most likely to encode the FA-AX biosynthetic enzymes. Subsequent work with GT61 family members involving gene suppression in wheat endosperm and heterologous expression of rice and wheat family members in Arabidopsis strongly suggest that GT61 family members are involved in xylan α-(1,3)-arabinofuranosyltransferase (XAT) activity (Anders et al., 2012). However, insertion mutants of rice *Os02g22380*, a GT61 family member, showed reductions in cell wall associated Xyl, exhibited a dwarf phenotype and, based on xylan structural analysis, suggested that the plants were defective in a ß-(1,2)-xylose–AX transferase activity (Chiniquy et al., 2012). Unexpectedly, these *Os02g22380* mutants also exhibited reductions in cell wall *p*CA and FA but not in Ara thus suggesting some role of the ß-(1,2)-xylose in either the modification of Ara by hydroxycinnamates or in the stability of coupled *p*CA/FA.

Many of the candidate grass gene approaches have relied on homology to Arabidopsis genes that were identified through analysis of plants altered in glucuronosylxylan biosynthesis including the *irregular xylem* (*irx*) mutants (Brown et al., 2007, 2009; Persson et al., 2007; Wu et al., 2009; Anders and Dupree, 2011). IRX10/IRX10L from the GT47 family and IRX9/IRX9L and IRX14/IRX14L from the GT43 family have been proposed to be responsible for xylan backbone elongation in Arabidopsis. Transposon insertion mutants of rice *Os01g70200*, a GT47 family member and ortholog to IRX10/IRX10L, exhibited a dwarf phenotype with a decrease in cell wall Xyl (Chen et al., 2013). *Os01g70200* mutants did have improved saccharification of cell wall carbohydrates without any alterations to lignin content. Activity studies with the IRX10 homolog from Physcomitrella patens demonstrated robust *in vitro* 1,4-XylT activity (Jensen et al., 2014) indicating its central role in xylan backbone synthesis.

Studies of four rice GT43 family members indicated a conservation of activity based on the functional complementation of Arabidopsis *irx9* and *irx14* mutants with select rice GT43 family members (Lee et al., 2014). The analysis of Lee et al. (2014) reiterated the presence of two functionally non-redundant classes of GT43 enzymes are needed for xylan production in Arabidopsis. Contemporary work from Ren et al. (2014) used site-directed mutagenesis of putative active site residues of GT43 family members IRX9/IRX9L and IRX14 to show that glycosyltransferase activity of IRX9/IRX9L is not needed for xylan biosynthesis in Arabidopsis. These results suggest a structural role for IRX9/IRX9L proteins in maintaining a protein complex catalyzing the xylan backbone. It is presumed that these results are conserved in monocots.

Members of the GT8 family of glycosyltransferase, GUX1, GUX2, and GUX4, are responsible for the addition of GlcA to xylans (Persson et al., 2007; Mortimer et al., 2010; Rennie et al., 2012) in Arabidopsis. Recent work has demonstrated unique, non-redundant xylan sequence domain specificities for GUX1 and GUX2 suggesting differential targeting of GlcA modifications to xylan sequence domains (Bromley et al., 2013). These latter results would suggest possible regulatory roles in controlling the positioning of xylan backbone modification and its influence on interactions with other cell wall components.

Biochemical approaches to identify enzymes involved in AX biosynthesis in grasses have been limited. Current evidence from all plant species strongly suggests that subcellular AX biosynthesis occurs within the lumen of the Golgi apparatus (Rennie and Scheller, 2014). Early *in vitro* enzyme activities using microsomal systems have been attempted to characterize intrinsic FA-AX associated glycosyltransferase activities (Porchia and Scheller, 2000; Kuroyama and Tsumuraya, 2001; Porchia et al., 2002; Zeng et al., 2008). Continuing the work of Zeng et al. (2008), research using complementary approaches including biochemical, proteomic, and transcriptomic analysis implicated three wheat glycosyltransferase proteins from the GT43, GT47, and GT75 families as candidates involved in AX biosynthesis (Zeng et al., 2010). The presence of GT43 and GT47 family members is consistent with work from Arabidopsis in assembly of the ß-(1,4)-xylose-xylan backbone. Interestingly, members of the GT75 family correspond to UDP-ß-L-arabinopyranose mutase (UAM), the enzyme responsible for the biosynthesis of UDP-ß-L-arabinofuranose (Konishi et al., 2007), were associated with the isolated complex. Immuno-purification of a detergent-solubilized wheat Golgi protein complexes exhibited glycosyltransferase enzyme activities consistent with XylT, (1,3)-AraT, and GlcAT activities however no direct proof was provided to link a specific catalytic activity with a specific polypeptide. Based on the author's work and others, the biosynthetic active sites for the AX glycosyltransferases is within the lumen of the Golgi. However, UAM proteins lack N-terminal signal sequences (Konishi et al., 2007; Rancour et al., 2015) and thus should be cytoplasmic: a result corroborated by the localization studies of Rautengarten et al. (2011) for the Arabidopsis orthologs. Therefore, the interaction between the lumenally-oriented GT43/GT47 and the cytoplasmic GT75 implies a mode of localizing the GT75/UAM to the cytoplasmic surface of the Golgi.

Recent work using Asparagus (*Asparagus officinalis* L.) as a model system for non-commelinid monocot xylan biosynthesis, has shown robust xylan biosynthesis and lignification activity post-harvest (Song et al., 2015). Taking advantage of these activities, RNA-seq was used to identify candidate genes involved in those activities. Further work using heterologous expression of asparagus genes and site-directed mutants have corroborated findings from Arabidopsis where (1) AoIRX9, AoIRX10, and AoIRX14 are core components of a Golgi-localized xylan synthesis protein complex, and (2) a functional glycosyltransferase active site is required for AoIRX10 and AoIRX14 catalytic activity (Zeng et al., 2016).

The subcellular organization of substrate biosynthesis and its utilization in xylan biosynthesis can provide a level of regulation previously underappreciated. Nucleotide sugars are believed to be the substrates for plant cell wall polysaccharide biosynthesis. The biosynthesis of the primary nucleotide-sugar substrates for GAX biosynthesis are metabolically related and are derived from sequential step-wise inter-conversions from UDP- α-D-Glc to UDP- α-D-GlcA to UDP- α-D-Xyl to UDP- β-L-Ara*p*, and, final to UDP- β-L-Ara*f* (Bar-Peled and O'neill, 2011). The enzymes involved in this metabolism are conserved in plants (Yin et al., 2011). The early work of identification and characterization of the inter-conversion enzymes occurred in Arabidopsis and suggested possible synthesis localizations both in the cytoplasm and the Golgi lumen depending on the involvement of a specific gene product. The consumption of nucleotide-sugars for GAX biosynthesis would be within the lumen of the Golgi. However, characterization of the rice UAM, the enzyme responsible for conversion of UDP-β-L-Arap to UDP-β-L-Araf is only found in the cytoplasm (Konishi et al., 2007; Rautengarten et al., 2011). Functional assessment of *Brachypodium* nucleotide-sugar inter-conversion enzymes in GAX biosynthesis via *in planta* RNAi indicated that the UAM was the most sensitive to gene expression alterations that resulted in cell wall composition changes (Rancour et al., 2015). Recent work in Arabidopsis has indicated that the cytoplasmic localized UDP-Xylose synthases (UXS) are responsible for providing more substrate for xylan biosynthesis than the Golgi localized family members (Kuang et al., 2016). These results suggest that transport of UDP- α-D-GlcA, UDP- α-D-Xyl, and UDP- β-L-Ara*f* into the Golgi are all needed for GAX biosynthesis and could function as control points for GAX biosynthesis. These results therefore suggest a need for transport of UDP- β-L-Ara*p* from the Golgi lumen to cytoplasm (Temple et al., 2016). No gene product nor direct evidence for this activity has been described.

FA-mediated cell wall crosslinking correlates with biomass recalcitrance and negatively impacts cell wall utilization (Hartley, 1990; Hatfield et al., 1999a,b; Buanafina, 2009). The mode of how FA and *p*CA are coupled to AX in terms of the identity of the substrates, the enzyme, and subcellular location of the relevant reactions is not well understood (Hatfield and Marita, 2010). Early work suggested that either FA-glucose (Obel et al., 2003) or FA-CoA (Yoshida-Shimokawa et al., 2001) could be the source for FA for FA-GAX synthesis. Using metabolic labeling time-courses, the rapid kinetics of GAX feruloylation suggested FA coupling to GAX occurs intracellularly (Mastrangelo et al., 2009). Current models suggest that select classes of BAHD acyltransferases (D'auria, 2006) are responsible for the FA- and *p*CA-esterification of GAX. BAHD transferases utilize acyl-CoA thioester substrates to catalyze the acylation of an acceptor nucleophile oxygen or nitrogen of a broad range of compounds. Work in rice and *Brachypodium distachyon* has further implicated select BAHD transferases in FA-AX biosynthesis (Piston et al., 2010; Bartley et al., 2013; Molinari et al., 2013). Piston et al. (2010) used RNAi approaches to suppress select BADH transferase expression resulting in reductions in cell wall associated ester-linked FA. Molinari et al. (2013) correlated BAHD transferase gene expression with cell wall FA and *p*CA content in *Brachypodium* to identify candidates responsible for FA-esterification of AX.

It is unclear what the significance of the ester-linked *p*CA modification of AX is since *p*CA does not readily form dimers when compared to FA (Weng et al., 2010). Recent work characterizing activation-tag rice lines of the “Mitchell” clade of BAHD transferases, a reference to the PF02458 gene clades identified by Mitchell et al. (2007), indicated that overexpression of OsAT10 resulted in cell wall FA reductions by 60% and an increase in AX-coupled ester-linked *p*CA by 300%. In addition, OsAT10 activation tagged plants did not exhibit any morphological defects or decreases in biomass production. Tagged-OsAT10 cell wall material had higher cell wall glucose content and improved saccharification but without any changes to lignin. These results suggest that high expression of OsAT10 can promote cell wall incorporation of *p*CA in place of FA, thus limiting cell wall crosslinking and improving cell wall digestibility. In addition, the results suggest that mechanisms are available to sense and respond to reduced FA crosslinking, and the stability granted by it, by increasing cell wall cellulose amounts. These results suggest that *p*CA substitution of AX could provide a mechanism to affect cell wall crosslinking capacity without drastic changes in the chemical nature of the substitution.

Though BAHD transferases have been implicated in FA and *p*CA esterification of AX, the mechanisms by which these occur is unclear. Proposed AX glycosyltransferase activity is confined to the lumen of the Golgi apparatus. However, the BAHD transferases implicated thus far in AX esterification contain no secretory pathway signal sequences or transmembrane domains and thus are expected to be cytoplasmic. Therefore, it is unlikely that direct acylation of the polymerizing AX occurs by these BAHD transferases and thus this poses a challenge to understand the subcellular topological context by which FA/*p*CA-AX is synthesized and subsequently trafficked to its site of cell wall incorporation.

The availability of nucleotide-sugar substrates can have significant consequences on Golgi-associated polysaccharide biosynthesis and FA-AX composition. Rice plant mutant in a nucleotide-sugar transporter exhibit cell wall composition alterations (Zhang et al., 2011). FA and *p*CA are ester-linked to the 5-OH of α-(1,3)-Ara*f* units. Mutant rice and *Brachypodium* plants with suppressed UDP-ß-L-Ara*p* mutase (UAM) gene expression exhibit decreased cell wall Ara*f*, FA and *p*CA derived from FA/*p*CA-AX (Konishi et al., 2011; Rancour et al., 2015). These observed phenotypes are due presumably to limiting the capacity to synthesize UDP-ß-L-Ara*f*, the α-1,3-Ara*f* T substrate. Rice and *Brachypodium* UAM proteins do not contain canonical secretory pathway signal or transmembrane sequences (Konishi et al., 2011; Rancour et al., 2015) and thus are predicted to be cytoplasmic, similar to the *Arabidopsis* orthologs (Rautengarten et al., 2011). Rautengarten et al. (2011) showed that Arabidopsis UAM isoforms that have enzymatic active also localize as peripheral Golgi membrane proteins. Knowing that rice UAM activity is contained within a large protein complex [~41 kDa monomer by SDS-PAGE but from rice tissue was ~460 kDa by size-exclusion chromatography (Konishi et al., 2007)] and assuming a conserved localization with the grass orthologs, sets up the tantalizing possibility that the mutase could be associated with the acyltransferase to allow for acylation of UDP-ß-L-Ara*f* to give UDP-ß-L-Ara*f* -FA as precursor to FA-AX synthesis and be positioned for direct transport into the Golgi for immediate use. These topological issues make FA-AX biosynthesis as much of a cell biology issue as a biochemical one. This model however has several unresolved issues. Firstly, having a nucleotide-sugar-hydroxycinnamate conjugate would require alterations in the conventional specificity of a nucleotide-sugar membrane transporter and a glycosyltransferase that would be unaffected by the additional ester-linked hydroxcinnamate. Transporter and glycosyltransferase proteins fitting these criteria have not been identified nor has UDP-ß-L-Ara*f* -FA or UDP-ß-L-Ara*f* -pCA intermediates. The lack of observable substrates could be simply down to a mechanism analogous to substrate channeling where (1) cytoplasm-oriented Golgi-associated UAM and acyltransferase generate UDP-ß-L-Ara*f*-FA or UDP-ß-L-Ara*f-p*CA intermediates, (2) the products are directly transported across the Golgi membrane by a specific transporter that is associated with the putative UAM/acyltransferase complex, and (3) the nucleotide-sugar-FA/*p*CA is directly consumed by the acyl-sugar-transferase which transfers the Ara*f* -FA/pCA to the xylan. The remaining uridine nucleotide would be recycled back to the cytoplasm (Abeijon et al., 1997) to complete the cycle.

An alternative mechanism for FA and *p*CA attachment to GAX in the Golgi lumen could be through unidentified Golgi transporters for CoA-FA and/or CoA-*p*CA, followed by transfer to accepters (Ara*f* already conjugated to the xylan backbone) within the Golgi. Acetyl-CoA transporters have been identified in other systems (i.e., Jonas et al., 2010) but it is unclear if these exist in plants (Schultink et al., 2015). This model would require signal sequences on FA/*p*CA transferases to properly localize them within the Golgi. One possible *Brachypodium* candidate could be Bradi2g23740 and its homologs. This gene exhibited an expression profile that mirrored cell wall ester-linked FA throughout development (Rancour and Hatfield, unpublished data) and is predicted to contain an N-terminal signal sequence consistent with entry into the secretory pathway. It remains to be determined whether Bradi2g23740 and its homologs are involved in FA conjugation to grass GAXs.

Although significant progress has been made in identifying protein factors that influence FA-AX biosynthesis, our understanding of how FA-AX is assembled is still lacking. It is clear that the molar ratios of Ara:Xyl and FA:Xyl change during development within grasses (Carpita, 1984a; Gibeaut and Carpita, 1991; Obel et al., 2002; Hatfield et al., 2008b; Rancour et al., 2012), however is this achieved during biosynthesis or post-synthesis processing? Are different α-1,3-Ara*f* T and α-1,2-Ara*f* T enzymes with altered processivity expressed developmentally or in a tissue-specific manner? Alternatively, do glycosidase activities, such as α-arabinofuranosidase, analogous to those observed in dicot plant systems (Goujon et al., 2003; Montes et al., 2008) have a role in grasses? Although these questions remain to be answered they point to areas of research that can be addressed.

## Organization of the cell wall

How the formation of lignin occurs within the cell wall matrix remains unresolved. Much is known about the synthesis of the monolignols and the phenolics, FA and *p*CA, but there are still gaps concerning how all the wall matrix components get organized into a functional cell wall. A recent review by Barros et al. (2015) highlights the complexity of the lignification process in higher plants. Even though the majority of work has been done on dicot and gymnosperm species compared to grasses there is valuable insight into possible processes. The question of transport of components into the cell wall is far from complete. There is good evidence that cell wall-associated proteins and select matrix polysaccharides including hemicelluloses and pectins are transported to the wall via exocytosis of Golgi derived vesicles (Li and Chapple, 2010). The mode of monolignol and acylated monolignol transport to the wall matrix remains unclear especially in grasses. There is conflicting evidence as to the involvement of glycosylation of monolignols in the transport process. There is evidence for the involvement of specific transporters (ATP-binding cassette like transporters) to move monolignols across the plasma membrane. These transporters may also utilize glucosylated monolignols and glucosidase activity once they are in the cell wall (Li and Chapple, 2010; Wang et al., 2013). Glucosylation of monolignols is an attractive process to help coordinate actual lignification within the wall space since radical formation could not occur with glucose attached. This in turn would allow accumulation of monolignols into a location, removal of the glucose followed by a radical mediated polymerization. A bulk type of reaction compared to a simple one on addition to produce the lignin polymer may explain the preponderance of β-O-4 type linkages (Touzel et al., 2003; Nakamura et al., 2006; Habrant et al., 2009). More importantly this should increase the efficiency of the lignification process creating a localization of lignin that is anchored by FA on GAX and initiated by the localization of peroxidase or laccase in the same area (Schuetz et al., 2014). Chapelle et al. (2012) have shown in Arabidopsis that monolignol glucosides appear to be involved in storage but not in the process of lignification. Similar work has not been done in grasses so extrapolating to a grass system may not be appropriate.

**Figure 4:**
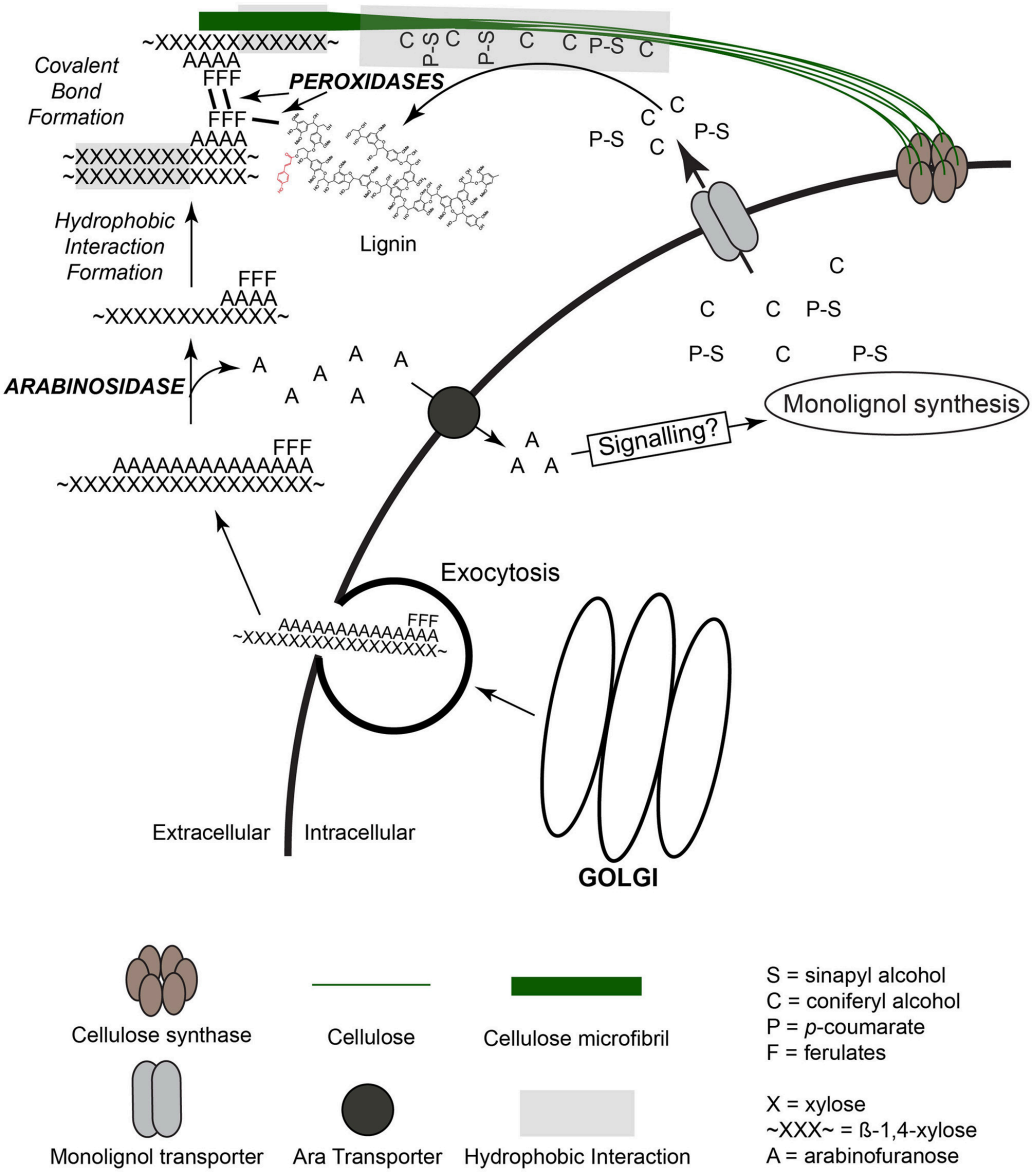
**Model for extracellular organization of cell wall synthesis and polymer organization**. Cellulose is synthesized by cellular membrane cellulose synthase complexes and forms stable microfibrils through hydrogen-bonding of individual cellulose polymers. Monolignols, synthesized within the cytoplasm, are transported to the extracellular space, associate via hydrophobic interactions with cellulose and/or xylans, and are coupled into lignin polymer through the action of wall peroxidases. Feruloylarabinoxylan (FA-AX) is synthesized within the Golgi and transported to the extracellular space by exocytosis. Once extracellular, activity of arabinofuranosidase removes arabinose residues to promote increased hydrogen-bonding capacity of xylan backbone with other xylans and cellulose. Ferulic acid dimer formation and coupling to lignin occurs during oxidative reactions coordinated with lignin polymerization.

It seems likely that the transport of monolignols and acylated monolignols in grasses is not a random process, but would be located close to sites of cellulose synthesis and GAX incorporation to build the matrix (Figure 4). The incorporation of matrix polysaccharides into the wall would precede the transport of monolignols. Initially during cell expansion only the matrix polysaccharides would be released to become associated with the cellulose. Ferulates attached to the GAX may help anchor the GAX into place as the matrix polysaccharides are being reprocessed to meet the functional needs of the wall. It is apparent there is coordination of these processes although there is no clear understanding of how this may occur. If GAX molecules are introduced into the wall free space in a highly substituted form perhaps the action of specific glycosidases would be needed for their integration. In addition, the released Ara and/or GlcA could act as signals to positively influence monolignol synthesis. Information regarding putative signaling pathways and possible transporters to recycle released Ara and/or GlcA is lacking. The role of sugar uptake transporters for Ara*f* and/or GlcA in grass cell wall development needs to be investigated at the gene level to identify genes and gene families that might be involved. In addition, a coordinated developmental study of gene expression combined with enzyme activity involving glycosidases would help establish what activities are critical and establish possible timeline of events. The difficulty with these studies is at any one point in time there may be over lapping stages of wall formation, i.e., wall initiation to secondary wall formation in grasses. In grasses such as corn or grain sorghum it would be possible to identify newly formed cell walls to more mature and lignifying walls all in the same internode. There are techniques for eluting enzymes from the apoplastic space of cell walls (a likely place for remodeling enzymes) to determine activity coordination with wall formation (Li et al., 1989; Lohaus et al., 2001; O'leary et al., 2014).

Though there are potential pathways for the movement of materials into the cell wall matrix it is unclear how the wall becomes organized. Once secreted, the movement of GAX molecules into the free space then into position to add to the wall matrix may play a pivotal role in final wall organization. Are these latter movements facilitated or dependent on the local physicochemical environment? Feruloylated GAX molecules may be synthesized in a coordinated fashion and post-synthesis modified to remove excess Ara units through the action of arabinofuransidase activity (Figure 3). The formation of feruloyl dimers between FA-GAX molecules suggests that FA is not randomly distributed along the xylose backbone. Rather two or three FA molecules may be attached to the arabinosyl side branches in close clusters along the xylan backbone and this is repeated over the entire molecule. This could be visualized as two or three ferulates clustered together followed by regions of 4–6 non-substituted arabinosyl units followed by another feruloylated cluster followed by a longer stretch of non-substituted arabinosyl units. Such a pattern would be repeated in the newly formed GAX molecules. It does not necessarily mean that the GAX has a specific molecular size and a ridged placement of ferulates along the xylan backbone. This arrangement would allow cross-coupling between two FA molecules of different xylans without the need to be perfectly aligned. The cross-coupling reaction would help to anchor the GAX polymers in place and in conjunction with the arabinofuranosidase activity promote hydrogen bonding to further tighten the wall matrix. It is likely that the appropriate oxidase (Laccase or peroxidase) would be positioned into the same general area to provide the oxidation potential to promote radical mediated cross-coupling of FA. Additional GAX polymers could be positioned and anchored as development continues. There is evidence that ferulates within the grass wall act as nucleation sites for lignin leading to localized formation into specific regions. Initially there may be multiple sites of lignification forming around FA nucleation points. During the early stages of lignification there would be small polymers of lignin that continue to expand as long as monolignols are supplied. The incorporation of laccases/peroxidases into the wall matrix close to the FA cluster would help control lignin formation since oxidation to form radicals could not occur without the addition of hydrogen peroxide thus providing another level of control over the process. As lignification continues these small sites of lignin may become connected together forming a much large lignin polymer. This may help explain why lignin in grasses are alkaline soluble especially in young tissues and decreases as the plant matures (Hatfield et al., 1994).

As already discussed the presence of *p*CA in the cell wall during lignification could possibly aid in lignin formation through improved oxidation of SA residues. Due to the electro-chemical properties of oxidized *p*CA, limiting radical coupling potential to cross-react with oxidized SA and CA residues, it may function as a radical shuttle system in the wall matrix. This would allow the formation of radicals on the growing lignin polymer. With FA acting as a nucleation site for lignin formation the *p*CA could assist in the continued formation of the lignin polymer from this attachment point in the wall. With *p*CA attachment via an ester linkage to monolignols, the incorporation of *p*CA-monolignol conjugate may act as a termination molecule for a developing lignin polymer. Having some *p*CA-monolignol conjugates exported along with individual monolignols would insure rapid oxidation and coupling. This action/process would keep the lignin polymer localized within a specialized region of the cell wall and allow for multiple areas of lignification within the cell wall. If more SA monolignols and SA-*p*CA conjugates, preferred conjugate formed in grasses, were shuttled out, this would lead to a molecule that is more linear in nature and less reticulated.

Why would termination of lignin polymers be an evolutionary advantage to grasses? It is known that grass lignin tends to be much more alkaline soluble than lignin found in typical dicots (Hatfield et al., 1994). This greater solubility suggests the extent of polymerization and/or the connectedness of grass lignin is less than in other species of plants. Furthermore, if free phenolics provide sites for alkaline and oxidative delignification resulting in increased alkali solubility of lignin (Lapierre et al., 1989; Froass et al., 1998), *p*CA attached to lignin as a termination molecule would help explain the solubility of grass lignin under basic conditions. Perhaps this serves grasses well especially during their development by helping to maintain a flexible structure. Because there is a need for lignin to provide additional strength to stems and leaves, it is counterproductive if these tissues actually become too rigid and subject plants to lodging especially during earlier stages of development.

The role of *p*CA or more appropriately the role of *p*-coumarate-monolignol conjugates, specifically the favored sinapyl alcohol- *p*CA conjugate (SA-*p*CA) is to help control the three-dimensional organization of grass lignin. It is known that syringyl type lignin (formed primarily from SA monolignols) forms a more linear structure (Kishimoto et al., 2005). This is not to say it forms a straight rod-like structure (such as cellulose) but does form a lignin polymer with little or no branching and with a lesser degree of polymerization (Figure 2). This is atypical of guaiacyl- or *p*-hydroxyphenyl-rich lignins formed by incorporation of coniferyl and *p*-courmaryl alcohol monolignols, respectively (Kishimoto et al., 2005). Although *p*CA could help facilitate lignin formation, once it is incorporated into the growing lignin polymer ester-linked as SA-*p*CA, it would terminate the lignification process until a new batch of monolignols were deposited into the wall.

The progress of lignification could be viewed as the establishment of nodes of lignin at nucleation sites with longer strands radiating out from these central concentrations of lignin in the cell wall matrix. If the strands are primarily syringyl types they most likely have few branch points (less reticulated) and possibly few if any covalent linkages between closely associated strands. Such a scenario would produce a structural matrix that could explain observations of both increased lignin solubility and increased cell wall digestibility as seen in grasses. Perhaps using visualization techniques such as click chemistry or fluorescence-tagged monolignols would help to define in plant lignin structure (Tobimatsu et al., 2013, 2014). In addition, grass cell walls tend to have a slower rate of structural carbohydrate degradation by ruminants though the extent is usually greater than the walls of dicot forages such as alfalfa (Galyean and Goetsch, 1993). It could be envisioned that over time during digestion the grass cell walls would be slowly degraded and as the degradation occurs the plasticity of the SA rich grass lignin would allow restricted access but sufficient flexibility to move some lignin strands out of the way creating easier access by ruminal microbes or released enzymes. Such an arrangement of lignin within the grass cell wall would explain why such a large portion of the lignin is soluble in hot acid detergent solutions. With limited cross coupling within grass lignin polymers the dissolution of matrix carbohydrates would allow additional solubilization of a portion of the lignin fraction.

## Summary

Controlling this process of cell wall assembly in grasses remains a question involving multiple steps in different metabolic pathways. It is proposed that ferulates play a central role in the organization as well as the function of grass cell walls. The role of ferulates to form dimers is critical for initial cell wall formation and expansion, but also aids in lignin formation within the wall matrix. The role of *p*-coumarate-monolignol conjugates could provide a means of controlling lignin size as well and influencing three-dimensional structure. The formation of FA-Ara substitutions on arabinoxylans is controlled by the activity of Ara*p* mutase and can act as a gating mechanism controlling the formation of Ara*f* and perhaps the level of substitution on the xylans and coupling of FA by feruloyl-CoA transferase to Ara*f*. Localization of oxidases (peroxidases and/or laccases) would occur at the lignin nucleation site i.e., FA and FA dimers must also be part of the coordinated lignification process.

## Author note

Mention of a proprietary product does not constitute a recommendation or warranty of the product by USDA and does not imply approval to the exclusion of other suitable products.

## Author contributions

RH has extensive research experience in grass cell wall chemistry and biochemistry and provided the main frame work and basic approach for this hypothesis article. DR was a Post-Doctoral fellow in the Hatfield lab and completed work in dealing with the characterization and molecular biology/biochemistry of structural carbohydrates in grass cell walls. JM (retired) was a research associate and has made many contributes to our increasing knowledge of grass cell wall chemistry and biochemistry.

## Funding

This work was supported by the U.S. Government, USDA-ARS.

### Conflict of interest statement

The authors declare that the research was conducted in the absence of any commercial or financial relationships that could be construed as a potential conflict of interest.

## Abbreviations

Ara*f*: arabinofuranose
AX: arabinoxylan
FA, ferulic acid: ferulates
GlcA: glucuronic acid
GAX: glucuronoarabinoxylan
*p*CA, *p*-coumaric acid: *p*-coumarates
Xyl: xylose.
